# Detection of Mesiobuccal Canal in Maxillary Molars and Distolingual Canal in Mandibular Molars by Dental CT: A Retrospective Study of 100 Cases

**DOI:** 10.1155/2010/291276

**Published:** 2010-06-02

**Authors:** Sushma Rathi, Jayaprakash Patil, Prashant P. Jaju

**Affiliations:** ^1^Department of Conservative and Endodontics, Sri Sai College of Dental Surgery & Hospital, Vikarabad, Andhra Pradesh 501101, India; ^2^Department of Oral Medicine, Diagnosis and Radiology, MGV's KBH Dental College & Hospital, Nashik, Maharashtra 422101, India

## Abstract

*Objective*. To detect presence of MB2 canal in maxillary molars and distolingual canal in mandibular molars by Dental CT. *Material and Methods*. A retrospective study of 100 Dental CTs was done. Axial and paraxial images obtained were used to assess the presence of MB2 canal in maxillary molars and distolingual canal in mandibular molars. *Results*. The youngest patient was of 11 years while the eldest patient was of 77 years. Males were 58 in number and females were 42 in number. MB2 canals were present in 57 patients and distolingual canal was present in 18 patients. Maximum MB2 canals were present in age group between 51 and 60 years, while distolingual canals were present in age group of 21–30 years. *Conclusion*. Dental CT allows adequate visualization of variation in root canal morphology and can be important diagnostic tool for successful endodontic therapy.

## 1. Introduction

The maxillary first molar is submitted to frequent endodontic treatment, and moreover, it presents the highest failure rates, often in relation to the presence of a second canal in the mesiobuccal root because the operator fails to detect, debride, or obturate this canal [1–4].

Similarly there is considerable variation in presence of distolingual canal in mandibular molars [1]. A thorough comprehension of the intricate 3-dimensional (3D) configuration of a root canal system is essential for the provision of successful endodontic therapy. Therefore the complex anatomic configuration of the maxillary first molar mesiobuccal (MB) root canal system has long been the subject of considerable scrutiny. The incidence of a second root canal in the mesiobuccal root (MB2) is 56.8%, in the distobuccal root 1.7%, and in the palatal root less than 1% [5, 6]. Frequency of MB2 canal have been investigated by numerous authors using different methods. In vivo clinical studies have included retrospective evaluations of patient records, radiographic assessments, and clinical examinations during endodontic treatment, both with and without the aid of magnification. In vitro laboratory studies have used extracted teeth and have included endodontic access, examination, radiography, scanning electron microscopy, grinding and sectioning, as well as numerous clearing studies using decalcification with India ink and other dyes [5, 7, 8]. Two-dimensional imaging like periapical radiography and panoramic radiography loses the battle for the lack of perception in the third dimension. With advent of Dental CT and Cone beam computed tomography (CBCT) the shortcomings of conventional imaging are overcome as they provide accurate and specific information. Dental CT is dedicated postscanning image evaluation software used for the teeth and the jaw, which creates panoramic and paraxial views of the upper and lower jaw. Dental CT provides adequate life-size visualization of osseous anatomy, with no streak artifacts of restorative material and a lower radiation dose compared with conventional CT. Literature reports studies on CT in endodontics for evaluation of additional canals [9–11]. There has not been any specific study on the incidence of detection of MB2 canal and distolingual canal by Dental CT. Hence this study was undertaken to assess the role of Dental CT in detection of additional canals.

## 2. Material and Methods

A retrospective study was conducted by examining Dental CT of 100 patients. These patients had undergone scanning for various treatment modalities especially pre-evaluation of implant sites. Scanning was performed at Jupiter Heart Scan Centre, Mumbai between the period of 2008-2009. The CT scan machine used was Seimens Somatom Sensation 64 which had multidetector technology with 32 detectors and 64 data channels. Scan direction was caudocranial beginning with the mandible base and extends to include the alveolar crest. Slice thickness of the images was adjusted to 1 mm with slice interval of 2 mm. Tube current is 90 mAs, tube voltage is 120, and table pitch is 0.9. Multiplanar reconstructions based on the dental CT protocol were obtained in the orthoradial and panoramic plane by using a dental software package (Syngo Dental CT 2006 A-W VB20B-W) on a workstation. The images were evaluated by two endodontists and a dental radiologist in a single session. Agreement was reached by means of a majority decision (at least two of three observers agreed). The present, number of MB2 canals in maxillary molars and distolingual canals in mandibular molars was evaluated. Statistical evaluation was done and the incidence of additional canals was determined.

## 3. Results

**Figure 1:**
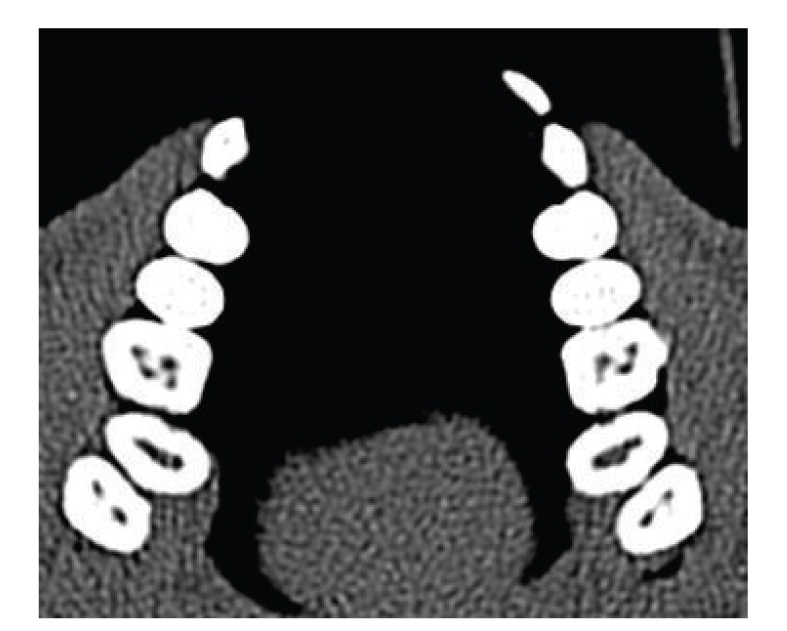
Axial view demonstrating MB2 in maxillary right first molar.

**Figure 2:**
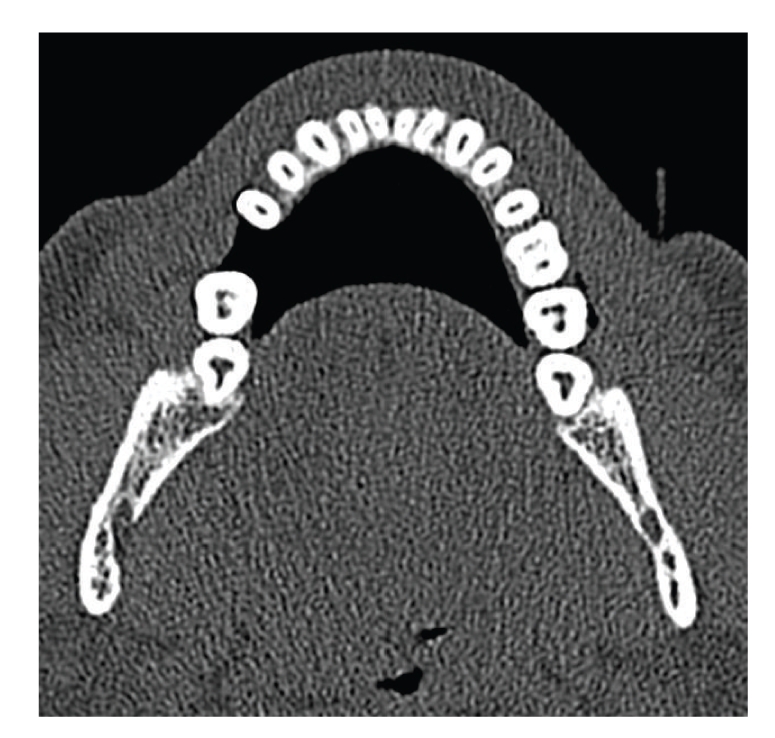
C-shaped canal in mandibular left second molar.

**Figure 3:**
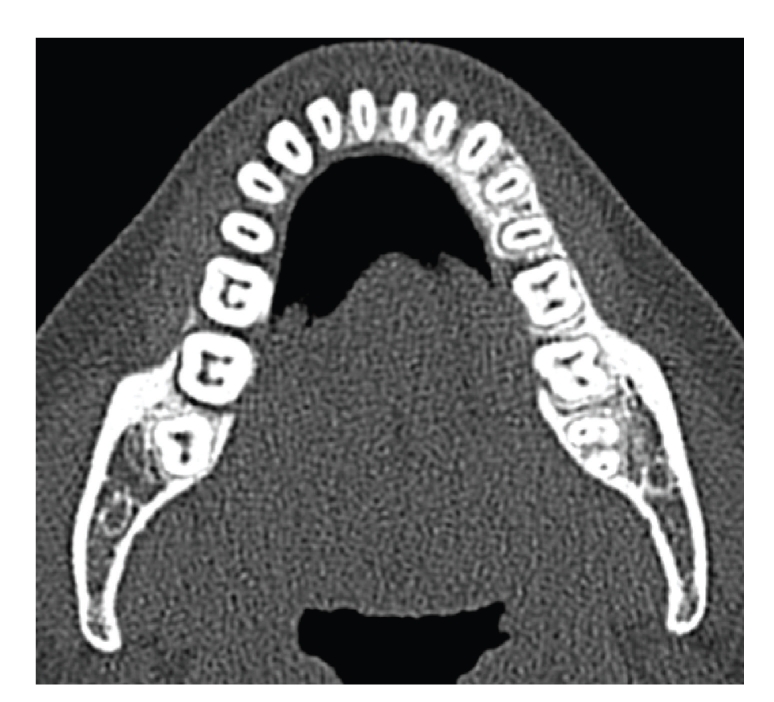
Axial view showing four canals in right first and second mandibular molars.

100 Dental CTs were evaluated for presence of additional canals. In total 58 males and 42 females patients were included in this study, with minimum age of 11 years and maximum age of 77 years. The average age of the patients was 44.41 years. Total 61 MB2 canals were detected in maxillary molars (Figure 1), and 18 distolingual canals were detected in mandibular molars (Figures 2 and 3). 39 MB2 canals were seen in maxillary first molar while 19 MB2 canals in maxillary second molar. 12 distolingual canals were detected in mandibular first molar and 6 canals in mandibular second molar (Table 1).

Table 1 shows age-wise distribution of canals. The highest MB2 canal incidence was detected in the age group of 51–60 years (29.50%), followed by 31–40 years and 21–30 years (19.67%), 41–50 years (16.39%), 61–70 years (8.19%), 10–20 years (6.55%).

The highest Distolingual canal incidence was detected of 21–30 years (52.63%), followed by 41–50 years (15.78%), 10–20 years and 31–40 years (10.52%), 51–60 years (5.26%).

**Figure 4:**
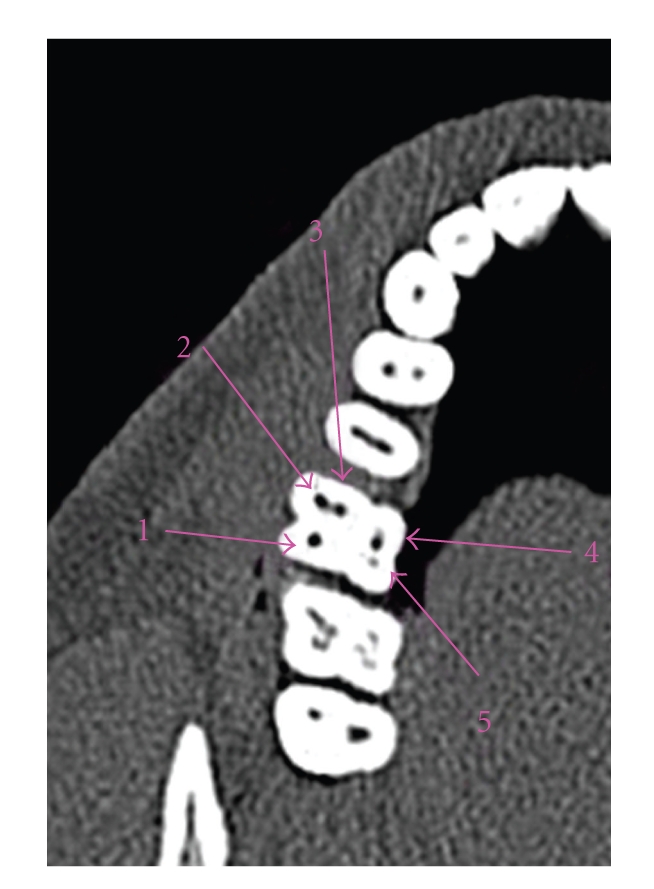
Maxillary molar with 5 canals.

There were 5 cases in which multiple MB2 canals were present in maxillary molars and one case having multiple distolingual canals. In one of our case there were 5 canals in right maxillary first molar and 6 canals in left maxillary first molar (Figure 4). Also in the same patient, four canals were seen in right and left mandibular molars.

## 4. Discussion

Variations in the root and root canal morphology, especially in multirooted teeth, are a constant challenge for diagnosis and management. The dentist needs to be familiar with the various root canal configurations and their variations for successful endodontic therapy.

Vertucci proposed a standardized method for categorizing known root canal anatomic variations, and a more clinically relevant classification of the root canal anatomy was described by Weine [12, 13]. However, there are many individual tooth variations and hence each case should be evaluated separately. Therefore, it is of utmost importance that all the canals are located and treated during the course of endodontic therapy. The literature describes wide variations in root canal morphology of maxillary first molars and mandibular molars [6]. The identification of the additional distolingual canals has been reported to be easily diagnosed compared with the MB2 canals which tend to be more elusive. It is generally accepted that the maxillary first molar has 3 roots and 3 canals with an MB2 canal seen in 56.8%–80.9% of the cases [5, 14]. However, there are many variations in canal number and configuration in maxillary first molar [5, 15]. The broad buccolingual dimension of the mesiobuccal root and associated concavities on its mesial and distal surface is consistent with the majority of the mesiobuccal roots having two canals while there is usually a single canal in each of the distobuccal and palatal roots [5]. 

 Mandibular molars also exhibit secondary root canals, over and above the traditional three. Although as many as five canals and as few as one and two canals rarely occur in mandibular molars, four canals are not unusual [16–18].

Dental CT has lately attracted considerable attention as a new diagnostic imaging technique in dentistry [10].

Dental CT has about eight times higher resolution than the medical CT. Therefore, this device is thought to be useful for the examination and diagnosis of hard tissues of the maxillofacial region including teeth, alveolar bone, and the jaws. Intraoral radiographs are a two-dimensional imaging modality of a three-dimensional structure. Hence anatomy in the third dimension cannot be assessed on radiographs. Because root canals tend to lie one behind the other in buccolingual plane, they get superimposed onto each other on periapical, panoramic radiographs and easily go undetected [19]. Dental CT is reformatting software used along with spiral/helical CT and allows assessment in all three dimensions. Hence, we undertook this new imaging modality to study the variation in anatomy of maxillary and mandibular molars and its role in endodontic treatment. Cleghorn et al. showed that there were 2 (multiple) MB canals in 57% of the teeth and only a single canal in 43% [5]. When the tooth shows only 1 buccal root, it is possible that the tooth has indeed only 1 buccal root or that the 2 buccal roots have fused. However, maxillary first molars with a single buccal root have not been described in the literature [15, 19]. The literature demonstrates extensive anatomical variations in the number of roots and canal morphology of maxillary first molars [5]. Stone and Stroner reported variations of the palatal root of maxillary molars such as a single root with 2 separate orifices, 2 separate canals, and 2 separate foramina; 2 separate roots, each with 1 orifice, 1 canal, and 1 foramen; and a single root with 1 orifice, a bifurcated canal, and 2 separate foramen [6]. The incidence of a maxillary first molar with 2 separate canals in the palatal root is less than 1% [6]. There are many studies corelating the variation in anatomy with respect to information on ethnic background, age, or gender. Walker reported on the root anatomy of maxillary first premolars, mandibular first premolars, and the high incidence of three-rooted mandibular first molars in Asian patients without reporting incidence of a second mesiobuccal canal (MB2) in the maxillary first molar [20–22]. Study by Weine et al. in Japanese population about incidence of MB2 was similar to the incidence reported for other ethnic backgrounds [2, 5]. Studies amongst the Asian populations have shown a greater tendency for a second canal in the distal roots of mandibular first molars compared with other populations [23].

One of the variations that can occur in mandibular first molars is radix entomolaris (RE). RE is a supernumerary distolingual root with various occurrences in different populations ranging from 3% of the African population to more than 30% of the mongoloid population [24].

As per our knowledge, no study has been conducted to determine the incidence of MB2 and distolingual canal by CT in Indian population. Age was found to have an effect on the incidence of MB2. Fewer canals were found in the MB root because of increasing age and calcification [25, 26]. In our study, age group between 51–60 years showed maximum MB2 cases while more of distolingual canals were present in age group of 21–30 years. The greater incidence of MB2 in 51–60 year age group could be because of more numbers of cases belong to this particular group, while no additional distolingual canal was observed in patients above the age group of 60 years. According to Sert and Bayirli gender and race were important factors to consider in preoperative evaluation of canal morphology for nonsurgical root canal therapy [27]. In our study there was not a significant difference in incidence of MB2 and distolingual canals according to sex of patients. There is a great deal of variation in results published on the incidence of MB2 and distolingual canals due to multiple factors like study design (clinical/laboratory), type of study, population studied and definition of what constitutes a canal which varied with individual authors [5].

The more common use of operating microscope or loupes in recent clinical studies has resulted in an increased prevalence of the clinical detection of the MB2 canal [28–30]. A study by Sempira and Hartwell found that the use of an operating microscope did increase the incidence of MB2 [29]. They attributed the lower incidence in their study to their characterization of a canal as one that must be negotiated and obturated to within 4 mm of the apex. Patient's radiation exposure to a conventional CT has been approximately 100–300 microsieverts (mSv) for maxilla and 200–500 mSv for mandible [31]. Nevertheless, the radiation exposure (for both maxilla and mandible) to CBCT has been 34–102 mSv, which has depended on the exposure time and scan resolution [32]. 

Dental CT has been proven to be superior over other diagnostic modalities by many studies in detection of anatomic variations [33, 34]. To increase the ex vivo assessment results, the association of a 3-dimensional technology and the operating microscope would be more appropriate because this technology could provide more details of the root canal system [35]. 

CT has been able to detect multiple canals in maxillary molars with maximum of 6 canals detected [36–38]. In one of our cases there were 5 canals in right maxillary first molar and 6 canals in left maxillary first molar. Also in the same patient, four canals were seen in right and left mandibular molars.

Nakata et al. studied the use of 3DX, a type of dental CTs and its role in endodontics. The major range of clinical applications mentioned by the authors included (a) observation of expansion of periradicular lesions of each root of multirooted teeth, (b) confirmation of the number, shape, and course of root canals, such as the mediopalatal root canal (MB2) of the maxillary first molar and gutter-shaped root canal of the mandibular second molar, (c) observation of 3D relationship between the periradicular region and the maxillary sinus or mandibular canal, (d) confirmation of the presence and position of fenestration, root fracture, root resorption, and perforation, (e) differentiation of the periradicular lesion from similar lesions, such as nonodontogenic cysts including nasopalatine cyst, and cementoma, and (f) measurement of the size of periradicular lesion and the distance between it and relational structures [39]. Thus, 3DX can be a useful diagnostic tool in endodontics as it provides a lower radiation dose with high resolution images. Stropko observed that by scheduling adequate clinical time, by using the recent magnification and detection instrumentation aids, and by having thorough knowledge of how and where to search for MB2, the rate of location can approach 93% in maxillary first molars [30].

## 5. Conclusion

In endodontic therapy, the quality and quantity of the information obtained from radiographic examinations is very important because it affects the diagnosis, treatment planning, and prognostic stability. In daily clinical practice, there are some cases where the conventional intraoral radiography and/or panoramic radiography alone does not provide enough information on the pathologic conditions, anatomical forms and structures, and positional relationships.

 Computed tomography (CT) in the form of Dental CT allows detailed three-dimensional (3D) observation of those forms and shapes, thus increasing the success rate of endodontic therapy.

## Figures and Tables

**Table 1:** Age-wise distribution of MB2 and Distolingual canals.

	10–20 years	21–30 years	31–40 years	41–50 years	51–60 years	61–70 years	71–80 years
No. of Patients	7	17	17	16	28	12	3
MB2 (First molar)	3	7	7	8	13	1	0
Second molar	0	5	4	2	5	3	0
Third molar	1	0	1	0	0	1	0
Total	**4**	**12**	**12**	**10**	**18**	**5**	**0**
Distolingual (first molar)	2	6	2	2	0	0	0
Second molar	0	4	0	1	1	0	0
Total	**2**	**10**	**2**	**3**	**1**	**0**	**0**

Total (MB2 + distolingual)	**6**	**22**	**14**	**13**	**19**	**5**	**0**
